# Autism Spectrum Disorder in Anorexia Nervosa: An Updated Literature Review

**DOI:** 10.1007/s11920-017-0791-9

**Published:** 2017-05-24

**Authors:** Heather Westwood, Kate Tchanturia

**Affiliations:** 1 Psychological Medicine, Institute of Psychiatry, Psychology and Neuroscience, King’s College London, London, SE5 8AF UK; 2 South London and Maudsley NHS Trust National Eating Disorders Service, Psychological Medicine Clinical Academic Group, London, UK; 3 Department of Psychology, Illia State University, Tbilisi, Georgia

**Keywords:** Anorexia nervosa, Autism spectrum disorder, Systematic review, Eating disorder, Female ASD

## Abstract

**Purpose of Review:**

There is growing interest in the relationship between anorexia nervosa (AN) and autism spectrum disorder (ASD). This review aimed to synthesise the most recent research on this topic to identify gaps in current knowledge, directions for future research and reflect on implications for treatment.

**Recent Findings:**

Eight studies assessing the presence of ASD in AN were identified in the literature along with three studies examining the impact of symptoms of ASD on treatment outcome. Research with young people and using parental-report measures suggest lower rates of co-morbidity than previous adult studies.

**Conclusions:**

The wide range of diagnostic tools, methodologies and populations studied make it difficult to determine the prevalence of ASD in AN. Despite this, studies consistently report over-representation of symptoms of ASD in AN. Co-morbid AN and ASD may require more intensive treatment or specifically tailored interventions. Future longitudinal research and female-specific diagnostic tools would help elucidate the relationship between these two disorders.

## Introduction

Anorexia nervosa (AN) is a severe eating disorder (ED) characterised by low body weight, intense fear or gaining weight and undue influence of weight and shape on self-evaluation [1]. It tends to manifest during adolescence [2] and has the highest mortality rate of any psychiatric disorder, with no gold standard treatment [3], high treatment dropout and relapse rates. In contrast, autism spectrum disorder (ASD) is a pervasive developmental disorder with marked difficulties with social interaction and communication and repetitive, stereotyped interests and behaviours (APA, 2013). For ASD to be diagnosed, symptoms must be present during infancy. While both AN and ASD are rare disorders within the general population, affecting around 0.3 and 1%, respectively [4, 5], they are associated with opposite gender ratios. AN is reported to be up to ten times more prevalent in females while the reported gender ratio for ASD is four males to one female [6]. Interestingly, in non-referred samples, there are only two to three males for every female with ASD [7] suggesting that many females are not referred and thus never receive a diagnosis [8].

Interest in the potential link between the two disorders began with suggestion that a shared underlying genetic vulnerability may interact with environmental factors to manifest as AN in girls during adolescence and ASD in boys during infancy [9]. Since then, growing research has attempted to elucidate the nature of the relationship between the two disorders, focusing on both elevated presence of ASD in AN and shared underlying difficulties in cognitive, social and emotional functioning [10–13].

Two previous reviews have examined the prevalence of ASD or elevated ASD symptoms in AN [14, 15]. Huke and colleagues [14] investigated the prevalence of ASD in ED populations, synthesising studies which used a variety of different diagnostic tools. The mean estimated prevalence of ASD from Huke and colleagues [14] review was 23%, suggesting an over-representation of ASD in EDs. However, six of the eight studies in Huke and colleagues’ review were based on longitudinal research with the same, Swedish community cohort [16–21]. Despite the diagnostic criteria for ASD requiring symptoms to be present during the early developmental period, the prevalence of estimated ASD ranged from 8 to 37% during the course of this longitudinal research, which used different diagnostic tools at each follow-up. This suggests that different assessment methods along with changing diagnostic criteria, e.g. the update from DSM-III-R to DSM-5 leads to unstable levels of reported ASD in AN populations. This review did not include studies with young people and concluded that studies from different cohorts were needed. Westwood and colleagues [15] conducted a systematic review and meta-analysis of studies using the self-report autism spectrum quotient AQ; [22] or abbreviated, ten-item version AQ-10; [23] to assess ASD symptoms in AN. Individuals with AN were found to have significantly more difficulties associated with ASD than healthy controls.

Despite the potential benefits of accurately assessing the prevalence of ASD in AN, doing so is challenging. Missed or delayed diagnosis of ASD in females during childhood could leave them vulnerable to the development of secondary mental health problems [24] including AN, which may obscure the identification of ASD when manifested as extreme rigidity or obsessive interest in calories or exercise [25]. The nature of the relationship between AN and ASD is complicated further by the possibility that some individuals without ASD may come to exhibit behaviours associated with the disorder during the acute phase of AN [26]. This trait versus state conundrum, along with the variety of different diagnostic criteria used and heterogeneous groups assessed makes it difficult to draw conclusions on whether ASD really is over-represented in AN, relative to the general population or other psychiatric disorders.

The aim of this review is to synthesise the current literature on the relationship between ASD and AN, including studies with young people and research examining the potential impact of ASD on treatment outcome. While there is growing evidence of similarities between the two disorders in terms of cognitive and socioemotional processing [12, 11, 13], studies focusing purely on such similarities are outside the scope of this review. While some studies have examined ASD across the spectrum of EDs, the majority have focused on its prevalence within AN. Therefore, only studies with participants with a diagnosis of AN are included, although where studies include more than one patient group, e.g. AN and bulimia nervosa (BN), this is stated. As self-report measures such as the AQ are limited to the assessment of current symptoms rather than accounting for any developmental history of ASD and rely on the subjective insights of the responder, which may be confounded by the acute phase of AN, studies which are based entirely on self-report questionnaires have been excluded from this review.

## Review of the Literature

### Studies Using Diagnostic Assessment Tools

Diagnostic guidelines for ASD [27] recommend using both developmental and observational assessment tools. This has led to studies assessing for ASD in AN using standardised diagnostic or screening tools including the Autism Diagnostic Observation Schedule, 2nd Edition ADOS-2; [28]; the Development and Well-being Assessment DAWBA; [29] and the Developmental, Dimensional and Diagnostic Interview 3Di; [30]. The ADOS-2 is a semi-structured assessment for ASD and is the most widely-used and best validated direct observational measure of characteristics associated with ASD [27]. The DAWBA is a self and informant-report screening measure, designed to generate DSM psychiatric diagnoses for children and adolescents. The 3Di is a validated parental report, diagnostic measure of ASD. It demonstrates good sensitivity with confirmed clinical diagnosis for DSM-5 [31]. Eight studies assessing ASD in ED populations using various clinical assessment tools were identified in the literature since the publication of Huke and colleagues’ review [14] and are summarised in Table 1.

**Table 1:** Studies using clinical assessments of ASD in individuals with AN

Study	N and diagnosis	Mean (SD) age	Assessment tool	Result
Pooni et al., (2012)	EOED =22 TD controls =24 ASD controls =20	13.0 (1.6) 13.0 (2.4) 11.6 (2.0)	3Di-sv	One (4.5%) participant with EOED had ASD. Elevated levels of ASD symptoms in EOED group.
Rhind et al., (2014)	AN =150	16.9 (2.13)	DAWBA	4% received possible or definite diagnosis of ASD
Mandy & Tchanturia (2015)	AN =7 EDNOS =2 BN =1	26.4 (6.59)	ADOS-2 Module 4	50% (pre-selected sample) scored above cut-off on ADOS-2.
Vagni et al., (2016)	AN =29 BN =25 BED =13	19.8 (1.0) 24.5 (1.6) 27.0 (1.9)	RAADS-R	33% of participant were classified as high levels of autistic symptoms.
Bentz et al., (2017)	AN =43 AN-*R* =28 TD controls =41	16.1 (1.5) 18.4 (1.6) 17.7 (2.2)	ADOS-2 Module 4	16% of individuals with AN and 21% of those with AN-R scored above cut-off on the ADOS-2.
Postorino et al., (2017)	AN =30 TD controls =35	14.19 (1.56) 13.6 (1.61)	ADOS-2 Module 3 or 4	10% of individuals with AN scored above cut-off on the ADOS-2.
Westwood et al., (2017)	AN =60	26.3(7.8)	ADOS-2 Module 4	23.3% scored above cut-off on ADOS-2.
Westwood et al., (in press)	AN =40	15.2 (1.52)	ADOS-2 Module 4 3Di-sv	52.5% scored above cut-off on the ADOS.2 10% scored above cut-off on both the ADOS-2 and 3Di-sv.

*ADOS-2* autism diagnostic observation schedule, 2nd edition, *AN* anorexia nervosa, *AN-R* anorexia nervosa, restrictive subtype, *ASD* autism spectrum disorder, *BED* binge eating disorder, *Bulimia Nervosa* bulimia nervosa, *DAWBA* development and well-being assessment, *EDNOS* eating disorder not otherwise specified, *EOED* early onset eating disorder, *N* number of participants, *RAADS-R* Ritvo autism Asperger diagnostic scale, revised, *3Di-sv* developmental, dimensional and diagnostic interview, short version

#### Studies with Adults

A small case series conducted by Mandy, Tchanturia [32] aimed to assess the feasibility of using the ADOS with an ED population. The study recruited a pre-selected sample of women EDs who had suspected ASD due to exhibiting social and flexibility difficulties and who did not respond to standard ED treatment protocols. Half of the women assessed met diagnostic criteria on the ADOS, while a further two were deemed likely to have ASD, despite not meeting the required cut-off on the ADOS. While the study demonstrated the utility of using the ADOS, the design of the study prevented conclusions being drawn about the prevalence of ASD.

This pilot study was extended by Westwood and colleagues [33•] who used the ADOS-2 to assess for ASD symptoms in a sample of 60 women diagnosed with AN. The proportion of participants who scored above cut-off on the ADOS-2, indicating the presence of symptoms characteristic of ASD, was the same as the mean prevalence rate (23%) reported in Huke and colleagues’ [14] systematic review. This is the largest study to date to use a “gold standard” diagnostic measure of ASD with an adult cohort of women with AN. Despite this, information on the developmental history of the participants was not obtained so conclusions regarding the aetiology of the symptoms, i.e. whether ASD was present prior to the onset of the disorder, could not be drawn. Elevated ASD symptoms were also associated with increased obsessive-compulsive symptoms and alexithymia. The presence of other co-morbid symptoms such as these could mediate the relationship between AN and apparent ASD. Alternatively, they could suggest that individuals with both AN and ASD are more likely to experience additional mental health problems.

To assess the heterogeneity of ASD symptoms in EDs, Vagni and colleagues [42] routinely screened all new outpatients, aged 15 and or over, admitted to a specialist ED ward using the Ritvo Autism Asperger Diagnostic Scale Revised RAADS-R; [43]. The aim of the study was to find a tool able to discriminate between individuals with high and low levels of autistic symptoms and to assess their relative prevalence. The RAADS-R was used as a clinical, structured interview and was chosen instead of the ADOS as the latter has been criticised for its ability to discriminate between adults with co-morbidities with no previous ASD diagnosis [44••] and requires extensive and specific training. Using this measure, 33% of individuals were classified as having elevated ASD symptoms. However, as the RAADS-R still relies on the subjective insights of the interviewee and no attempt to ascertain developmental history from relatives was made, it is not possible to determine with certainty whether any ASD symptoms predated the onset of the ED.

#### Studies with Young People

To control for the impact that starvation and duration of AN may have on the presence of ASD symptoms, recently published studies have recruited younger people with either early onset EDs or individuals with short illness durations. To investigate whether scores on the ADOS-2 are corroborated by parental report, Westwood and colleagues [34••] assessed a sample (*n* = 40) of adolescent females, aged 12 to 18. Twenty-one (52.5%) scored at or above cut-off on the ADOS-2 so their parents were asked to complete the 3Di, short version 3Di-sv; [35], a well-validated measure of ASD which provides categorical diagnoses as well as measuring dimensionally-occurring ASD symptoms across both clinical and typically developing populations. Only 4 (10%) individuals scored above cut-off on both the ADOS-2 and 3Di-sv, thus meeting research criteria for ASD. These results suggest that while a significant proportion of young people present with symptoms associated with ASD, these symptoms were not present during the early developmental period, a requirement for ASD diagnosis. Alternatively, it could be that such symptoms are not recognised by parents during infancy. Other studies conducted with young people with AN have reported similar findings when using parental-report measures [36, 37].

Pooni and colleagues [36] attempted to address the issue of separating the presence of current ASD symptoms and a developmental history of ASD in individuals with early onset ED (EOED). While EOED is difficult to define [38], the study adopted criteria operationalised by Nicholls and colleagues [39] including: weight loss or failure to gain weight; fear of weight gain and preoccupation with gaining weight. When ICD-10 criteria were used, 20 of the 22 participants were assigned a diagnosis of AN or atypical AN. Using the 3Di, Pooni and colleagues [36] found that a diagnosis of ASD was no more likely in individuals with EOED than in typically developing controls. However, the EOED group did exhibit elevated levels of ASD symptoms. A similar finding was reported by Rhind and colleagues [37] who used the DAWBA. Only 4% of female participants were assigned a possible diagnosis of ASD while up to 39% of participants showed difficulties with social aptitude and peer relationships.

Two other studies with young people have utilised the ADOS-2 to assess the presence of observable symptoms associated with ASD. Postorino and colleagues [40•] assessed a group of female adolescents (*N* = 30), diagnosed with AN during the acute phase of illness. Contrary to Westwood and colleague’s findings, only 10% scored above cut-off on the ADOS-2. While a standardised developmental measure such as the 3Di was not used, according to a clinical interview, no participant was given a diagnosis of ASD. The other study [41••] aimed to compare social functioning in participants with first-episode, recent onset (within 12 months of participating) AN with those recovered from the disorder. Despite a previous diagnosis of ASD being ruled out, 16 and 21% of individuals respectively scored above cut-off on the ADOS-2. Similar levels of functional impairment between the two groups suggest that social difficulties are not limited to the acute phase of the illness. As the study did not investigate the developmental history of these difficulties, the findings were limited to examination of social difficulties rather than ASD per se.

The limited evidence available from studies with young people suggests that despite individuals who are currently ill with AN displaying high levels of symptoms characteristic of ASD, the history of these symptoms is not corroborated by parental report. There are several possible reasons for this, including but not limited to: (1) such symptoms are an epiphenomenon, arising from the ill-state associated with AN; (2) parents under-report or do not recognise the behaviour of their child as being associated with ASD, particularly in girls, as ASD is often considered to be a “male disorder”; (3) The diagnostic tools being used are not sensitive to detecting ASD symptoms in a predominantly female, AN population. Pooni and colleagues [36] also suggest that the developmental trajectory of EDs into adulthood may account for some of the discrepancy in ASD prevalence between young people and adults. The prognosis of adolescent onset EDs is relatively good [42] but individuals who do not recover will likely be seen in adult services. The presence of ASD or elevated ASD symptoms may therefore be a predictor of poorer treatment outcome, leading to its relative over-representation in adult services.

## Nationwide Cohort Study

One study [43] aimed to investigate the co-morbidity of ASD and AN and familial aggregation of the disorders by using Danish registers to identify individuals classified as having AN, atypical AN, infantile autism, atypical autism or Asperger syndrome who had records for either inpatient or outpatient care for that disorder. To study familial aggregation of the disorders, probands and their parents and siblings diagnosed with EDs, ASD, major depression or any psychiatric disorders were included. Five thousand and six individuals with AN and 12,606 with ASD were identified through screening. Probands with a primary diagnosis of AN had a highly elevated risk of receiving a diagnosis of ASD, with the risk for males being higher than for females. However, there was an even higher risk of being diagnosed with ASD in individuals whose primary diagnosis was major depression. A family history of AN was associated with an elevated risk of ASD but this risk was comparable to that seen in families with a history of major depression and any psychiatric disorder. This suggests that while there is increased risk of co-morbidity of AN and ASD and aggregation of ASD in families with AN, the relationship between the two disorders is non-specific. Thus, ASD may increase the likelihood of receiving any psychiatric diagnosis but not specifically a diagnosis of AN.

### Summary of Literature Review

There is consistent evidence, using a variety of assessment tools, that symptoms associated with ASD are over-represented in EDs, particularly in AN. Studies report between 4 and 52.5% of participants meeting suggested clinical cut-off for ASD. Despite this, research utilising parental reports to determine the presence of such symptoms during early childhood report much lower rates [36, 37, 34••] of participants meeting clinical cut-off. While the aetiology of these symptoms remaining unclear, three further studies have examined the potential impact of ASD symptoms on treatment outcome in AN. These studies are discussed in the next section of this review.

### Impact of ASD Symptoms on Treatment Outcome

To date, three studies, displayed in Table 2, have attempted to examine the potential impact of ASD symptoms on treatment outcome in ED groups using clinical measures of ASD.

**Table 2:** Studies assessing the impact of ASD on treatment outcome in individuals with AN

Study	Aim	N and diagnosis	Mean (SD) Age	Assessment tool	Outcome measure	Results
Nielsen et al., (2015)	Examine trends and effects of co-existing ASD on outcome in AN	AN =51	32	Asperger syndrome diagnostic interview; checklist for autistic disorder and Asperger’s syndrome; AQ or a combination	MROAS	11.8% classified as ASD. The presence of ASD contributed to restricted outcomes in mental, psychosexual and socio-economic state. The more diagnostic stability of ASD, the worse the outcome.
Tchanturia et al., (2016)	Examine response to group CRT in patients with high and low autistic symptoms	AN =35	26.2 (7.7)	ADOS-2; AQ; AQ-10 or a combination	DFlex; MR; patient feedback questionnaire	40% of participants scored above cut-off for ASD symptoms on at least one measure. Patients with elevated ASD symptoms showed no significant improvements on any outcome measure
Stewart et al., (2017)	Assess levels of ASD symptoms in girls with restrictive EDs; examine treatment outcome in girls with elevated ASD symptoms	AN =250 EDNOS-R/OSFED =159	14.6 (1.76)	AQ; DAWBA; SAS	MROAS	286 girls completed all treatment outcome measures. 20/289 (6.9%) scored above 30 on the AQ. 52/338 (15.4%) scored below 16 on the SAS. No evidence of increased ASD-related developmental concerns as measured by the DAWBA. Treatment as usual did not result in different physical outcomes for girls with ASD symptoms but they had a greater need for treatment augmentation.

*ADOS-2* autism diagnostic observation schedule, 2nd edition, *AN* anorexia nervosa, *AQ* autism spectrum quotient, *AQ-10* autism spectrum quotient, short version, *CRT* cognitive remediation therapy, *DAWBA* development and well-being assessment, *DFlex* detail and flexibility questionnaire, *ED* eating disorder, *EDNOS-R* eating disorder not otherwise specified, restrictive, *MR* motivational ruler, *MROA*S Morgan-Russell outcome assessment schedule, *OSFED* other specified feeding or eating disorder, *SAS* social aptitude scale

The largest study [44••] used clinical audit data to examine the impact of ASD symptoms on treatment outcome in girls referred to a specialist ED service. Again, the presence of current ASD symptoms was elevated in this cohort but there was no evidence of raised prevalence of childhood ASD, as measured by the DAWBA. Girls with elevated ASD symptoms, as measured by the AQ required greater augmentation of treatment reflected in admission to an intensive day treatment programme or inpatient wards. Scores on the Social Aptitude Scale SAS; [45], a parental-rated measure of a young person’s social skills, were not related to treatment augmentation. Less change in the self-report EDE-Q subscale scores of weight concern, shape concern and global score was also associated with higher DAWBA ASD scores.

In the fourth follow-up of the longitudinal Swedish cohort study described in the introduction to this review, Nielsen and colleagues [46] examined the effect of ASD on outcome using the Morgan-Russell outcome assessment schedule MROAS; [47]. The cohort were initially assessed at age 16 then again at 6-year, 10-year and 16-year follow-ups. The tools used to diagnose ASD differed across all four studies, consisting of the following: study (1) structured interview with mother; study (2) Dewey social awareness test, checklist for autistic disorder and Asperger’s syndrome; study (3) Asperger Syndrome Diagnostic Interview, checklist for autistic disorder and Asperger’s syndrome and study (4) Asperger Syndrome Diagnostic Interview, checklist for autistic disorder and Asperger’s syndrome and the AQ. Six AN participants were classified having ASD at all four assessment points. The MROAS is a structured interview, concerned with the clinical features of AN. Responses to questions about eating, weight, mental state and attitudes yield five sub-scores, used to monitor change in clinical status [48]. The presence of ASD contributed to restricted outcomes in mental, psychosexual and socio-economic state, as measured by the MROAS: the more diagnostic stability of ASD, the worse the outcome.

The impact of symptoms associated with ASD on specific treatment outcome has been examined by Tchanturia and colleagues [49] in relation to group Cognitive Remediation Therapy (CRT). In this cohort study, ASD was assessed with at least one of the following: ADOS-2, AQ or AQ-10 depending on whether a trained researcher was available to administer the ADOS-2 to at the time of admission. Fourteen (40%) participants scored above cut-off on at least one measure, suggesting the presence of elevated ASD symptoms. The Detail and Flexibility Questionnaire [50] was used to assess self-reported changes in cognitive rigidity and attention to detail. Whereas the low-scoring group showed significant improvement in cognitive rigidity and self-reported ability to change following the group CRT intervention, the high-scored ASD group showed no improvements on any outcome measure. While other co-morbidities such as anxiety and depression were not accounted for, this data suggests that elevated ASD symptoms may be associated with poorer response to existing ED treatments, specifically targeted at improving difficulties with flexibility, associated with ASD [11].

### Assessment of ED Symptoms in ASD

Despite numerous studies exploring the presence of ASD in AN populations, little research has examined the opposite phenomenon, i.e. EDs in people with ASD. While there is scientific consensus supporting an association between ASD and food selectivity [51], the latter includes food refusal, limited repertoire of foods and is thus not necessarily associated with ED psychopathology. There have been case reports of girls with an underlying diagnosis of Asperger syndrome exhibiting symptoms of AN [52–54], emphasising the importance of diagnosing AN in individuals in ASD so that appropriate treatment can be sought. The inclusion of Avoidant and Restrictive Food Intake Disorder (ARFID) in DSM-5 [1], characterised by limited consumption of food due to its sensory characteristics or past negative experiences with food, has led to research into the presence of ARFID in ASD [55]. There is also evidence that ARFID is common in young people with eating disorders. One study found that 22.5% of young people attending a day treatment programme for EDs met ARFID criteria [56]. Higher rates of co-morbid anxiety disorders, learning disabilities and pervasive developmental disorders (including ASD) were found in the ARFID group, compared to those with AN, BN or OSFED.

Gillberg [57] recently coined the term ESSENCE (Early Symptomatic Syndromes Eliciting Neurodevelopmental Clinical Examinations) to cover neurodevelopmental disorders such as ASD and Attention Deficit Hyperactivity Disorder (ADHD) which often co-occur and lead to children presenting in clinical settings with difficulties in several domains of functioning. One study [58] aimed to examine the prevalence of EDs and eating pathology in a sample of 228 young adults and adults with ESSENCE. Within the entire sample, 7.9% had a current or previous ED but individuals with a diagnosis of ADHD more often affirmed eating pathology than individuals with ASD. This study suggests that EDs may be over-represented in individuals with neuropsychiatric disorders but not specifically ASD. Interestingly, the gender ratio in this study was not as skewed (1:2.5 male: female) as in the general population, with a large proportion of men experiencing EDs or reporting ED pathology.

## Future Directions and Clinical Implications

Diagnosing ASD in individuals with AN is a complex process which requires thorough clinical assessment including direct observation of symptoms and comprehensive developmental history. While attempts have been made to elucidate the relationship between ASD in AN, the presence of other co-morbidities, varying diagnostic tools and criteria, and the possibility of a distinct female autism phenotype make it difficult to draw firm conclusions about the true prevalence of ASD in AN. Recent studies have utilised developmental measures and recruited younger participants in an attempt to control for the impact that ED pathology may have on ASD symptoms. Despite this, studies have used different cut-off points to define elevated symptoms or assessment tools which may not be sensitive enough to distinguish ASD in females or differentiate between ASD and other co-morbidities.

Despite growing evidence that ASD presents differently in females than in males (e.g. [59]), standard diagnostic tools such as the ADOS-2 and Autism Diagnostic Interview-Revised, ADI-R; [60] and DSM-5 diagnostic criteria [1] have not reflected on these gender differences. As most diagnostic tools are validated with males, females are less likely to present with symptoms detected with such tools, leading to an underestimation of ASD in females [61]. Until routine assessment tools recognise and reflect the gender differences in ASD, diagnosing ASD in individuals with AN, who are not only predominantly female but who often present with a multitude of co-morbidities, will remain problematic. Future research focusing on the development of gender-specific diagnostic algorithms and screening tools is urgently needed.

Once gender-sensitive diagnostic tools have been established, further research utilising a longitudinal design and following stringent diagnostic criteria will be needed to fully understand the relationship between the two disorders. To determine whether undiagnosed ASD really does leave females at increased risk of developing AN, it would also be beneficial to identify “high risk” cases, e.g. sisters of individuals with ASD or young females with a diagnosis of high-functioning ASD and to assess for eating disorder psychopathology. To date, research has not focused on the lived-experience of individuals with both AN and ASD. If, as previously hypothesised [25], ASD can manifest as extreme rigidity or interest in calories, exercise or food, it may be that the ED pathology of individuals with ASD is qualitatively distinct from individuals with AN alone. Given that ARFID rather than AN per se may be more common in individuals with ASD, clinicians treating individuals with both an ED and elevated ASD symptoms should be mindful of the possibility that the individual is experiencing a distinct and different ED.

Regardless of the aetiology of ASD symptoms, the presence of such symptoms in AN has been associated with poorer treatment outcome and the need for more intensive treatment. While these symptoms may not be associated with a specific neurodevelopmental disorder, social and non-social difficulties characteristic of ASD may prevent individuals from engaging in conventional treatments and be associated with other symptoms such as depression, anxiety or OCD. Screening for ASD symptoms may therefore be beneficial, although clinicians should be cautious in formally diagnosing ASD without involvement from specialist ASD services. Controlled studies, comparing treatment response in individuals with and without ASD symptoms are also needed to determine whether specific treatment adaptations or care pathways are warranted.

## Conclusions

Despite differing assessment tools and recruitment from heterogeneous patient groups, studies consistently report elevated ASD symptoms in AN populations. It remains unclear whether these symptoms represent an underlying neurodevelopmental disorder, are exacerbated by ED pathology or whether they are mere similarities in cognitive and socioemotional functioning, also shared with other psychiatric diagnoses. Elevated ASD symptoms are often not corroborated by developmental history, but until a time when diagnostic tools account for gender differences in ASD, assessing the prevalence of ASD in AN will remain difficult. Regardless of the aetiology of these symptoms, individuals with social and non-social difficulties characteristic of ASD may require treatment adaptations or more intensive care. There is a need for robust longitudinal studies using thorough, gender-specific ASD assessment methods to further elucidate the relationship between the two disorders.

## References

[1] APA (2013). Diagnostic and statistical manual of mental disorders.

[2] Micali N, Hagberg KW, Petersen I, Treasure JL. The incidence of eating disorders in the UK in 2000–2009: findings from the General Practice Research Database. BMJ Open. 2013;3:e002646. doi:10.1136/bmjopen-2013-002646. 10.1136/bmjopen-2013-002646 PMC3657659 23793681

[3] National Institute for Health and Clinical Excellence. Eating disorders: core interventions in the management of anorexia nervosa, bulimia nervosa and related eating disorders. British Psychological Society; 2004. 23346610

[4] Hoek HW (2006). Incidence, prevalence and mortality of anorexia nervosa and other eating disorders. Current opinion in psychiatry.

[5] Brugha T, Cooper SA, McManus S, Purdon S, Smith J, Scott FJ et al. Estimating the prevalence of autism spectrum conditions in adults: extending the 2007 adult psychiatric morbidity survey. In: care TICfhas, editor. NHS; 2012.

[6] CDC. Prevalence of autism spectrum disorders—Autism and Developmental Disabilities Monitoring Network, United States, 2008. Morbidity and Mortality Weekly Report 2012. p. 1–19. 22456193

[7] Kim YS, Leventhal BL, Koh Y-J, Fombonne E, Laska E, Lim E-C, et al. Prevalence of autism spectrum disorders in a total population sample. Am J Psychiat. 2011; 10.1176/appi.ajp.2011.10101532 21558103

[8] Bargiela S, Steward R, Mandy W (2016). The experiences of late-diagnosed women with autism spectrum conditions: an investigation of the female autism phenotype. J Autism Dev Disord.

[9] Gillberg C (1983). Are autism and anorexia nervosa related?. The British journal of psychiatry: the journal of mental science..

[10] Oldershaw A, Treasure J, Hambrook D, Tchanturia K, Schmidt U (2011). Is anorexia nervosa a version of autism spectrum disorders?. Eur Eat Disord Rev.

[11] Westwood H, Stahl D, Mandy W, Tchanturia K. The set-shifting profiles of anorexia nervosa and autism spectrum disorder using the Wisconsin Card Sorting Test: a systematic review and meta-analysis. Psychol Med. 2016:1–19. doi:10.1017/S0033291716000581. 10.1017/S0033291716000581 27109830

[12] Davies H, Wolz I, Leppanen J, Fernandez-Aranda F, Schmidt U, Tchanturia K (2016). Facial expression to emotional stimuli in non-psychotic disorders: a systematic review and meta-analysis. Neurosci Biobehav Rev.

[13] Westwood H, Lawrence V, Fleming C, Tchanturia K (2016). Exploration of friendship experiences, before and after illness onset in females with anorexia nervosa: a qualitative study. PLoS One.

[14] Huke V, Turk J, Saeidi S, Kent A, Morgan JF (2013). Autism spectrum disorders in eating disorder populations: a systematic review. European eating disorders review: the journal of the Eating Disorders Association..

[15] Westwood H, Eisler I, Mandy W, Leppanen J, Treasure J, Tchanturia K. Using the autism-spectrum quotient to measure autistic traits in anorexia nervosa: a systematic review and meta-analysis. J Autism Dev Disord. 2015; doi:10.1007/s10803-015-2641-0. 10.1007/s10803-015-2641-0 PMC4746216 26542816

[16] Rastam M (1992). Anorexia-nervosa in 51 Swedish adolescents—premorbid problems and comorbidity. J Am Acad Child Adolesc Psychiatry.

[17] Gillberg IC, Rastam M, Gillberg C (1995). Anorexia-nervosa 6 years after onset. 1. Personality-disorders. Compr Psychiatry.

[18] Nilsson EW, Gillberg C, Rastam M (1998). Familial factors in anorexia nervosa: a community-based study. Compr Psychiatry.

[19] Nilsson EW, Gillberg C, Gillberg IC, Rastam M (1999). Ten-year follow-up of adolescent-onset anorexia nervosa: personality disorders. J Am Acad Child Adolesc Psychiatry.

[20] Rastam M, Gillberg C, Wentz E (2003). Outcome of teenage-onset anorexia nervosa in a Swedish community-based sample. European child & adolescent psychiatry..

[21] Anckarsater H, Hofvander B, Billstedt E, Gillberg IC, Gillberg C, Wentz E (2012). The sociocommunicative deficit subgroup in anorexia nervosa: autism spectrum disorders and neurocognition in a community-based, longitudinal study. Psychol Med.

[22] Baron-Cohen S, Wheelwright S, Skinner R, Martin J, Clubley E (2001). The autism-spectrum quotient (AQ): evidence from Asperger syndrome/high-functioning autism, males and females, scientists and mathematicians. J Autism Dev Disord.

[23] Allison C, Auyeung B, Baron-Cohen S. Toward brief “red flags” for autism screening: the short autism spectrum quotient and the short quantitative checklist for autism in toddlers in 1,000 cases and 3,000 controls [corrected]. J Am Acad Child Adolesc Psychiatry 2012;51(2):202–212 **e7**. doi:10.1016/j.jaac.2011.11.003. 10.1016/j.jaac.2011.11.003 22265366

[24] Yaull-Smith D. Girls on the spectrum. Communication National Autistic Society. 2008.

[25] Lai M-C, Baron-Cohen S (2015). Identifying the lost generation of adults with autism spectrum conditions. The Lancet Psychiatry.

[26] Hiller R, Pellicano E (2013). Anorexia and autism—a cautionary note. The Psychologist.

[27] NICE. Autism: recognition, referral, diagnosis and management of adults on the autism spectrum. National Institute for Health and Clinical Excellence; 2012.

[28] Lord C, Rutter M, DiLavore PC, Risi S, Gotham K, Bishop SI (2012). Autism diagnostic observation schedule, second edition (ADOS-2) manual (part 1) modules 1–4.

[29] Goodman R, Ford T, Richards H, Gatward R, Meltzer H (2000). The development and well-being assessment: description and initial validation of an integrated assessment of child and adolescent psychopathology. Journal of child psychology and psychiatry, and allied disciplines.

[30] Skuse D, Warrington R, Bishop D, Chowdhury U, Lau J, Mandy W (2004). The developmental, dimensional and diagnostic interview (3di): a novel computerized assessment for autism spectrum disorders. J Am Acad Child Adolesc Psychiatry.

[31] Slappendel G, Mandy W, van der Ende J, Verhulst FC, van der Sijde A, Duvekot J (2016). Utility of the 3Di short version for the diagnostic assessment of autism spectrum disorder and compatibility with DSM-5. J Autism Dev Disord.

[32] Mandy W, Tchanturia K. Do women with eating disorders who have social and flexibility difficulties really have autism? A case series. Molecular autism. 2015;6 doi:10.1186/2040-2392-6-6. 10.1186/2040-2392-6-6 PMC4459459 26056560

[33] • Westwood H, Mandy W, Tchanuria K. Clinical evaluation of autistic symptoms in women with anorexia nervosa. Molecular autism. 2017; doi:10.1186/s13229-017-0128-x. **This is the largest study to use the ADOS-2 to assess symptoms of ASD in women with AN.** 10.1186/s13229-017-0128-x PMC5356303 28331571

[34] •• Westwood H, Mandy W, Simic M, Tchanturia K. Assessing ASD in adolescent females with anorexia nervosa using clinical and developmental measures: a preliminary investigation. J Abnorm Child Psychol. 2017; doi:10.1007/s10802-017-0301-x. **This is the first study to use both the ADOS-2 and 3Di-sv to assess both observable and historical symptoms of ASD in adolescents with AN.** 10.1007/s10802-017-0301-x PMC5770498 28417276

[35] Santosh PJ, Mandy WP, Puura K, Kaartinen M, Warrington R, Skuse DH (2009). The construction and validation of a short form of the developmental, diagnostic and dimensional interview. European child & adolescent psychiatry..

[36] Pooni J, Ninteman A, Bryant-Waugh R, Nicholls D, Mandy W (2012). Investigating autism spectrum disorder and autistic traits in early onset eating disorder. The International journal of eating disorders.

[37] Rhind C, Bonfioli E, Hibbs R, Goddard E, Macdonald P, Gowers S (2014). An examination of autism spectrum traits in adolescents with anorexia nervosa and their parents. Molecular autism..

[38] Bravender T, Bryant-Waugh R, Herzog D, Katzman D, Kriepe RD, Lask B (2010). Classification of eating disturbance in children and adolescents: proposed changes for the DSM-V. European eating disorders review: the journal of the Eating Disorders Association.

[39] Nicholls DE, Lynn R, Viner RM (2011). Childhood eating disorders: British national surveillance study. The British journal of psychiatry: the journal of mental science..

[40] • Postorino V, Scahill L, De Peppo L, Fatta LM, Zanna V, Castiglioni MC, et al. Investigation of autism spectrum disorder and autistic traits in an adolescent sample with anorexia nervosa. J Autism Dev Disord. 2017; doi:10.1007/s10803-016-3023-y. **This study compared ASD symptoms in individuals with AN and healthy controls using the ADOS-2.** 10.1007/s10803-016-3023-y 28120263

[41] Bentz M, Jepsen JRM, Pedersen T, Bulik CM, Pedersen L, Pagsberg AK (2017). Impairment of social function in young females with recent-onset anorexia nervosa and recovered individuals. J Adolesc Health.

[42] Steinhausen HC, Boyadjieva S, Griogoroiu-Serbanescu M, Neumarker KJ (2003). The outcome of adolescent eating disorders: findings from an international collaborative study. European child & adolescent psychiatry.

[43] Koch SV, Larsen JT, Mouridsen SE, Bentz M, Petersen L, Bulik C, et al. Autism spectrum disorder in individuals with anorexia nervosa and in their first- and second-degree relatives: Danish nationwide register-based cohort-study. The British journal of psychiatry: the journal of mental science. 2015; doi:10.1192/bjp.bp.114.153221. 10.1192/bjp.bp.114.153221 25657359

[44] •• Stewart CS, McEwen FS, Konstantellou A, Eisler I, Simic M. Impact of ASD traits on treatment outcomes of eating disorders in girls. European eating disorders review: the journal of the Eating Disorders Association. 2017; doi:10.1002/erv.2497. **This study examined treatment augmentaiton and outcome in young people with AN and elevated symptoms of ASD.** 10.1002/erv.2497 28058799

[45] Liddle EB, Batty MJ, Goodman R (2009). The social aptitudes scale: an initial validation. Soc Psychiatry Psychiatr Epidemiol.

[46] Nielsen S, Anckarsater H, Gillberg C, Gillberg C, Rastam M, Wentz E (2015). Effects of autism spectrum disorders on outcome in teenage-onset anorexia nervosa evaluated by the Morgan-Russell outcome assessment schedule: a controlled community-based study. Molecular autism.

[47] Morgan HG, Russell GFM (1975). Value of family background and clinical features as predictors of long-term outcome in anorexia-nervosa: 4-year follow-up study of 41 patients. Psychol Med.

[48] Morgan H, Hayward A (1988). Clinical assessment of anorexia nervosa. The Morgan-Russell outcome assessment schedule. Br J Psychiatry.

[49] Tchanturia K, Larsson E, Adamson J (2016). How anorexia nervosa patients with high and low autistic traits respond to group Cognitive Remediation Therapy. BMC psychiatry.

[50] Roberts ME, Barthel FM, Lopez C, Tchanturia K, Treasure JL (2011). Development and validation of the Detail and Flexibility Questionnaire (DFlex) in eating disorders. Eat Behav.

[51] Mari-Bauset S, Zazpe I, Mari-Sanchis A, Llopis-Gonzalez A, Morales-Suarez-Varela M (2014). Food selectivity in autism spectrum disorders: a systematic review. J Child Neurol.

[52] Dudova I, Kocourkova J, Koutek J (2015). Early-onset anorexia nervosa in girls with Asperger syndrome. Neuropsychiatr Dis Treat.

[53] Rothery DJ, Garden GM (1988). Anorexia nervosa and infantile autism. The British journal of psychiatry: the journal of mental science.

[54] Fisman S, Steele M, Short J, Byrne T, Lavallee C (1996). Case study: anorexia nervosa and autistic disorder in an adolescent girl. Journal of the American Academy of Child & Adolescent Psychiatry.

[55] Lucarelli J, Pappas D, Welchons L, Augustyn M (2017). Autism spectrum disorder and avoidant/restrictive food intake disorder. Journal of developmental and behavioral pediatrics: JDBP.

[56] Nicely TA, Lane-Loney S, Masciulli E, Hollenbeak CS, Ornstein RM (2014). Prevalence and characteristics of avoidant/restrictive food intake disorder in a cohort of young patients in day treatment for eating disorders. Journal of eating disorders.

[57] Gillberg C (2010). The ESSENCE in child psychiatry: early symptomatic syndromes eliciting neurodevelopmental clinical examinations. Res Dev Disabil.

[58] Karjalainen L, Gillberg C, Rastam M, Wentz E (2016). Eating disorders and eating pathology in young adult and adult patients with ESSENCE. Compr Psychiatry.

[59] Mandy W, Chilvers R, Chowdhury U, Salter G, Seigal A, Skuse D (2012). Sex differences in autism spectrum disorder: evidence from a large sample of children and adolescents. J Autism Dev Disord.

[60] Lord C, Rutter M, Le Couteur A (1994). Autism diagnostic interview-revised: a revised version of a diagnostic interview for caregivers of individuals with possible pervasive developmental disorders. J Autism Dev Disord.

[61] Beggiato A, Peyre H, Maruani A, Scheid I, Rastam M, Amsellem F, et al. Gender differences in autism spectrum disorders: divergence among specific core symptoms. Autism research: official journal of the International Society for Autism Research. 2016; doi:10.1002/aur.1715. 10.1002/aur.1715 27809408

